# Selective modifications at the different positions of cyclodextrins: a review of strategies

**DOI:** 10.3906/kim-1910-43

**Published:** 2020-04-01

**Authors:** Jia Yue LIU, Xiao ZHANG, Bing Ren TIAN

**Affiliations:** 1 School of Pharmacy, Ningxia Medical University, Yinchuan P.R. China; 2 Pingliang Center for Disease Control and Prevention, Pingliang P.R. China; 3 College of Chemistry and Chemical Engineering, Xinjiang University, Urumchi P.R. China

**Keywords:** Cyclodextrin, modification, synthesis, application

## Abstract

Cyclodextrins (CDs) are natural, nontoxic, and biodegradable macrocyclic oligosaccharides. As supramolecular hosts, CDs have numerous applications in many aspects. However, nonsubstituted CDs have the disadvantages of solubility, unspecific recognition sites, and weak interactions with guest molecules. Therefore, new CD-based derivatives are successfully designed, synthesized, and widely used in various fields. This contribution outlines the research progress in CD derivatives. In particular, this review emphasizes the synthesis and application of CDs modified through functionalization in definite positions, random substitution, and reconstruction of the skeleton. At the end of this review, a summary and future directions are presented.

## 1. Introduction

**Figure 1:**
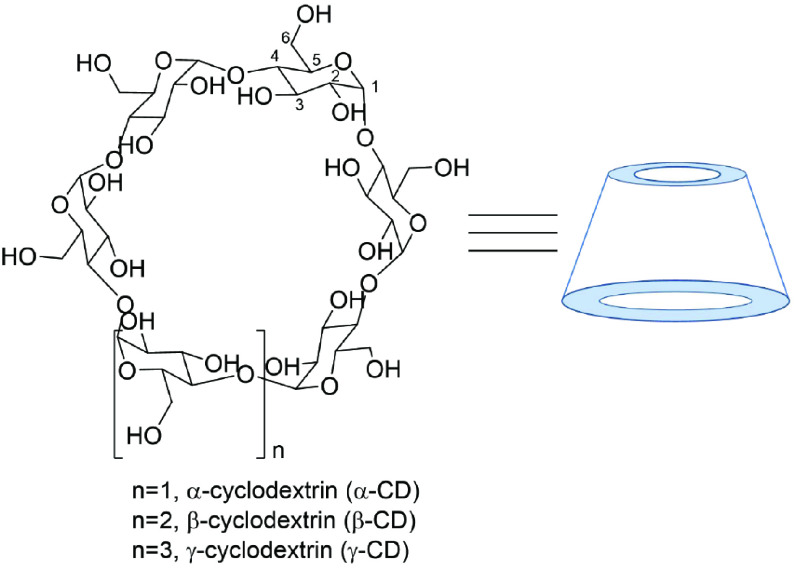
The structure and conformation of α-, β -, and γ -CD. Carbon atoms are named 1, 2, 3, etc. as usual.

Cyclodextrins (CDs) are distinguished cyclic oligosaccharides derived from the enzymatic reactions of starch (Figure 1) [1,2], which could be classified into three natural categories [3,4]. CDs that could be produced on an industrial scale are nontoxic and biodegradable [5,6]. Due to their special cavity structure, solubility, and cost, these oligosaccharides have been widely used. In particular, the hydrophobic cavity of CDs enables them to encapsulate other small molecules and generate inclusion complexes [7], which demonstrate host– guest relationships [8,9]. This remarkable property of encapsulation is very useful for guest molecules applied in many industries such as medicine [10,11], cosmetics [12–14], agrochemistry [15], supramolecular chemistry [16–18], enzymology [19,20], chromatography [21,22], and catalysis [23–27].

Currently, CD-based derivatives have attracted widespread interest as new hosts, and a considerable amount of effort has been made to modify CDs and improve their properties. Hydroxypropyl-, sulfobutylether-, and carboxymethyl-type β -CDs are versatile derivatives used to improve the solubility, stability, and physical properties of CD–guest complexes. The foremost strategy is to convert hydroxyl groups to other improved functional groups. Hence, modifications of CDs offer numerous opportunities and challenges for researchers [28–32]. Although Khan et al. [33] described the methods for modification of CDs, few studies have been conducted on the application of such derivatives. Much attention has been paid to the use of CD derivatives in material and medicine industry, but very few reports are available about the methods for modifying CDs [34,35]. Furthermore, new developments and methods, particularly the reconstruction of CD skeletons, have been revealed for the modification of CDs. Therefore, this review mainly focuses on the modifications and classifications of different positions of CDs while also analyzing the properties and practical applications of corresponding derivatives.

## 2. Inclusion complexes of CD derivatives

Since the discovery of CDs, their cavities have served as important tools to form complex compounds with other guest molecules. In macromolecular self-assembly, Chen and Jiang [36] summarized the process of inclusion complex formation. On the one hand, Tan et al. [37] determined the kinds of functional CD-based supramolecular assemblies and hydrogels when the CD inclusion complexes were applied in biomedicine. On the other hand, scholars have focused on the functional application of CD polymers with host–guest recognition, dynamic and adaptive materials, and other properties [38–40].

The host–guest system has been used in medicine, materials science, and several directions at this stage, and many new applications will become available.

Recently CDs have a wide range of applications in cancer treatment owing to the particularity of their different ring spaces. CDs could increase the solubility of anticancer flavonoids, such as curcumin and quercetin, which are very poorly soluble in water [41,42]. A novel CD complex of curcumin had superior attributes compared with free curcumin for cellular uptake and for antiproliferative and antiinflammatory activities, preferentially aggregating in the pancreas and possessing strong anticancer activity against pancreatic or tubulin surface cancer cells [41,43]. Similarly, the solubility of quercetin could be improved linearly with the increasing molality of CDs at the same temperature [44].

## 3. Selective modifications of CDs at definite positions

### 3.1. Monosubstitution of CDs

**Figure 2:**
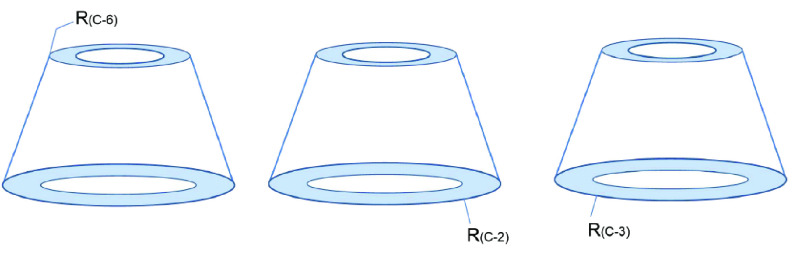
The three types of CD monosubstituted derivatives.

When natural CDs are unsuitable for a particular application, chemists begin to modify CDs to allow their derivatives to perform their intended function. To pursue an increasingly perfect structure, the idea of monosubstituted CD derivatives was conceived. However, the direct synthesis of single-isomer CD derivatives is challenging [45]. A single substitution has three possible isomers (Figure 2).

In recent years, the immobilization of CDs onto magnetic nanoparticles has drawn considerable attention on account of the unique size and physical properties of nanomaterials [46]. β -CD has been selectively functionalized and then immobilized into Fe_3_O_4_ nanoparticles. The magnetic properties of β -CD nanoparticles easily provide separation of enantiomers with respect to reducing the effort required for such separations. Moreover, the immobilization of CDs onto iron oxide magnetic nanoparticles provides a surface diversity associated with enhancing the stability of the CD [47].

#### 3.1.1. Two-position modification

**Figure 3:**
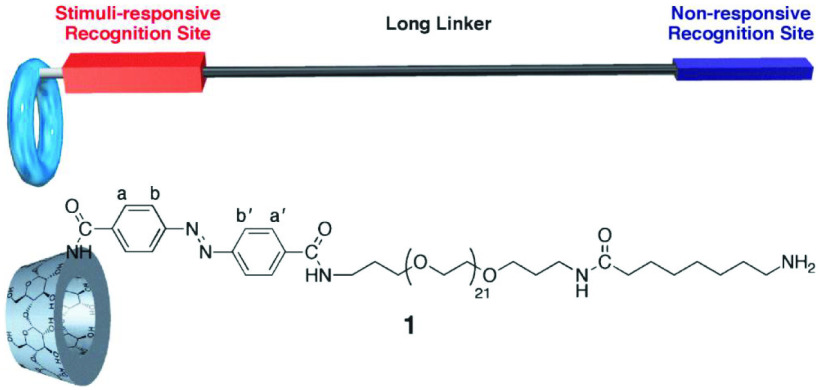
Molecular design and structure of α-CD derivative. Copyright 2010, Chemical Social of Japan.

The second side of CDs has two different types of hydroxyl groups to ensure that the space is crowded. Many articles have shown that the C-2 hydroxyl group seems to be easier to modify than the C-3 hydroxyl group. Li et al. designed and synthesized a new α-CD derivative linked by an oligo (ethylene glycol) chain that had two groups regulated by light (Figure 3) [48]. This structure of the derivative also causes a remarkable change in the hydrodynamic radii of the duplex dimer. The terminal amine can be used to modify the derivative and change its macroscopic dimension [42]. For the other type of CD, azobenzene was modified using β -CD. Researchers found that forming the desired product caused competition under several reaction conditions and reported measures to improve the reaction [49].

A few decades ago, chemists found some CDs with poor water solubility. To improve solubility, researchers began to transform the structure of CDs. 2-Hydroxy-β -CD that was modified by propylene oxide exhibited satisfactory water solubility. Complexes with testosterone, progesterone, and estradiol exhibited remarkable absorption effects. These complexes could also avoid the rapid first-pass loss in in vivo experiments. For β -CD, its 2,6-dimethyl derivatives and nonionic detergents were ineffective for buccal absorption. Hydrophilic β -CD complexed with hormones leads to ineffective gastrointestinal absorption. Further studies should be conducted on the role of 2-hydroxy-β -CD in the slow release of buccal tablets [50]. Zhao et al. successfully obtained two different types of 2-O-(2-hydroxybutyl)-β -CD and used them as chiral selectors to separate racemic drugs. The newly formed derivatives have better separation than natural β -CDs and another derivative (2-HP-β -CD) [51].

Given that hydroxyl groups are easily oxidized into other substances, researchers have developed a partial oxidation reaction to form carbonyl-β -CD. The solubility of the derivative was substantially enhanced because the intramolecular hydrogen bond network of β -CD was broken by the oxidation reaction. In addition, the novel β -CD derivative could generate an inclusion complex with ferrocene. Furthermore, the complex has effective electrical conductivity. The new combination of metal and CDs can be used in biosensor systems [52].

Liu et al. obtained two C-2 derivatives, which were modified by organoselenium. Then they used the method of circular dichroism and NMR to investigate their conformation. Furthermore, these derivatives had good enantioselectivity [53]. A previous work used copper sulfate as a catalyst to obtain a new β -CD derivative [54]. Zgani et al. developed a new method for synthesis of β -CD derivative with modified C-2 position. The interaction of host and guest was determined by many technologies when hydrocinnamic and adamantine carboxylic acids were used as guest molecules [55]. Meanwhile, a γ -CD derivative, which was modified with pyrene carbonyl in DMF, exhibited remarkable excimer emission and formed a stable inclusion complex with 1-borneol [56].

#### 3.1.2. Three-position displacement

Selective derivatization at C-3 is more difficult than that at C-2 hydroxyl groups due to the weaker acidity of the former. Only a few studies have been conducted on three-modified CDs [57–59].

**Figure 4:**
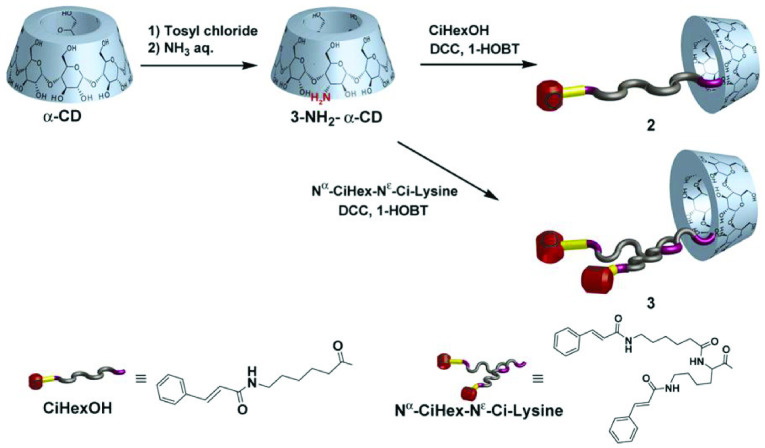
The structure of α-CDs; 2 has a cinnamamide moiety and 3 has two cinnamamide moieties. Copyright 2008, Elsevier.

Many researchers have made notable contributions in recent years. Miyawaki et al. successfully prepared branched supramolecular polymers. Some compounds formed linear polymers, and others formed hyperbranched polymers (Figure 4). The behavior of the branched polymers consisting of the complex of derivatives was investigated. The mixture of derivatives showed polymer-like properties. This phenomenon could be ascribed to the formation of a hetero-type inclusion complex and hydrophobic interactions and/or hydrogen bond [60]. A similar reaction was applied to successfully prepare β -CD derivative and obtained 30% isolated yield and greater than 90% regioselectivity. The cinnamyl group could be easily changed to multiple other groups, such as Ac and H. 2D NMR technology was applied to confirm and explain the substitution pattern in single-modified CDs [61].

3-Monoamino-α-permethylated CD and 3-monoamino-β -permethylated CD were synthesized using a new method described as the per-O-methylation of amino-CDs. In particular, the common group of tertbutyloxycarbonyl was used to protect the amino group because it was stable in the reaction condition and can be removed easily [62]. Masurier et al. designed and prepared an inclusion complex of β -CD with copper(II) in an aqueous medium; some of the products were supplied to markets [54].

Martina et al. developed a novel approach to synthesize three different CD types of 3I -deoxy-3I - azido derivatives and obtained pure altrose isomers; the use of US- and MW-assisted procedures proved very advantageous in terms of yield, reaction time, and product purity [63]. Suzuki et al. successfully synthesized a γ -CD derivative by three-point modification with pyrene carbonyl in DMF and studied its inclusion ability [56].

#### 3.1.3. Six-position displacement

**Figure 5:**
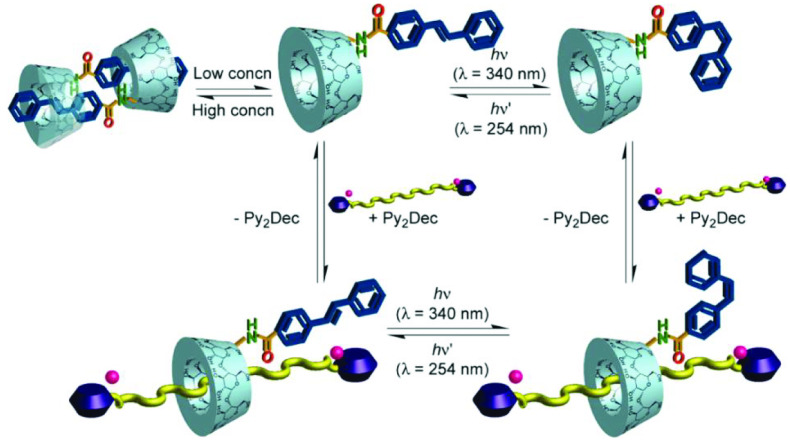
The two isomers of 6-stilbene-amide-α-CD and different inclusion behaviors. Copyright 2011, American Chemical Society.

C-6 hydroxyl groups have no effect on other hydroxyl groups and can be used to easily perform structural transformations. Sallas et al. firstly synthesized and obtained high yields of thio-β -CD derivatives by using the direct Mitsunobu reaction; they also explored further use of the method [64]. The majority of CD derivatives are utilized in chromatographic analysis. A new α-CD derivative was modified using a polyethylene glycolbased sol-gel method. The derivative used as the new stationary phase displayed remarkable column efficiency and satisfactory ability to separate aromatic isomers [65]. CD derivatives (β - or γ -chirasil-dex) were used as chiral stationary phases (CSPs) to separate enantiomers. Li et al. evaluated mono-6-deoxy-benzimide-β -CD enantioseparation and chiral recognition ability. Many analytes with rigid structures were separated by mono- 6-deoxyp-henylimine-β -CD [66]. Wang et al. successfully synthesized four cationic β -CD derivatives, which were used as novel CSPs by coating them onto porous spherical silica gel. The performance of these CSPs was examined using different types of alcohol. The derivative exhibited high separation ability upon different analyses [67]. Four other cationic β -CD derivatives were investigated in terms of enantioseparation ability, and the unsubstituted pyCDCl had the best resolving ability. Most analytes could be resolved using this chiral selector [68]. Wang et al. utilized click chemistry to modify mono-azido-β -CD derivatives with silica. The new derivative was used as a novel CSP and exhibited high stability and prominent enantioseparation effects [69]. Two isomers of 6-stilbene-amide-α-CD had interesting behavior; the stilbene moiety of the CD derivative could change between the axle and trans- or cis-6-StiNH-α-CD when forming inclusion complexes with an alkyl chain bearing pyridinium end caps (Figure 5). Similar findings were reported in previous works [70–72].

Lopez et al. reported a class of novel cup-shaped α-CD derivatives. The derivative containing an aldehyde substituent showed more effective catalysis ability than other analogues [73].

Le et al. designed and synthesized two β -CD derivatives; NMR and computer modeling were used to interpret data and the results showed that the triazole moieties had limited freedom of rotation to maintain the rigid and compact structure of the derivatives. In water-soluble experiments, the derivatives were higher in water solubility than the parent of β -CD. The MTT assay revealed that derivatives had no effect on cell viability under experimental conditions (1 mM). The solubility of prednisolone increased with increasing concentrations of derivatives [74]. Water-soluble amphiphilic anion receptors were successfully designed based on urea-substituted β -CDs, and the final products were stable [75]. Chitosan was used to functionalize β -CD to form a new product that had improved effect against inflammation-related diseases [76].

Yoon et al. synthesized β -CD and 6-deoxy-6-formyl-β -CD derivatives by using the Nace reaction. As the starting material, monoaldehyde was reacted with NaBH_4_, NaHSO_3_, NH_2_OH, NH_2_NH_2_, H_2_O, and PhNH_2_ with NaCNBH3 to form new β -CD derivatives, particularly the carboxylic acid of 6-deoxy-6-carboxy-β -CD; this work provided a general method for forming tosyl derivatives of β -CD [77]. Under mild detachment conditions, new C-6-monosubstituted CD derivatives were obtained by solid phase method [78]. Martina et al. found that regioselective synthesis of different functionalized CDs can be effectively performed under ultrasonic condition or microwave radiation [63].

When β -CD is reacted with R-configuration groups, the derivatives function as CSPs. Other researchers separated some chiral aromatic compounds by using CD derivatives. Molecular docking simulation was utilized to explore the recognition mechanism. S-2-Naphthalene methanol possessed greater coactions with R-CPGCD and R-HMPGCD than the R-isomer. The substituent derivative of R-CPGCD and the CD cavity were conducive to the discrimination of the S-isomer, but only the derivative group of R-HMPGCD played a dominant role in isolation. The R- and S-isomers in the R-HMPGCD system might have a high resolution value [79]. By using novel derivatives as CSPs, Li et al. evaluated the separation of many chiral compounds. The derivatives were characterized and prepared as chiral monolithic stationary phases [80].

Nanospheres could improve the water stability of camptothecin and display sustained release of the profile. It was found that a novel derivative possessed a special cavity, as determined using some technologies, such as transmission electron microscopy. The hollow nanosphere showed excellent drug-loading performance [81,82].

Many pathogens are formed during infection of the transmembrane pore of target cells. As important virulence factors, bacteria and viruses are commonly used as new therapeutic targets. Karginov et al. hypothesized that compounds could be designed to prevent pores and inhibit virulence factors; the opportunities for finding high-affinity blockers increased if they had the same symmetry with the target aperture. In this regard, several β -CD derivatives were synthesized to inhibit the action of lethal anthrax toxin (LeTx). By hole-blocking artificial lipid membranes and PA α-HL ion conduction ability, this method could provide a reference to find new and effective therapies using pore-forming proteins as a virulence factor of various pathogens [83].

Liu et al. synthesized CD derivatives. In 2005, they found a simple method for synthesis of water-soluble CD derivatives and modified them by using C60 . The derivative displayed desirable DNA-cleaving ability under the experimental conditions [84]. A new β -CD derivative modified by cyclohexylamine had high yield. The binding ability of the derivative with many naphthalene derivatives was also explored. Hence, this modification method improved the original binding ability of β -CD, except that of 2-naphthalene sulfonic acid sodium. Moreover, pH and salt concentration can influence the stability of the inclusion complex [85]. Two types of β -CD derivatives were satisfactorily obtained. Liu et al. characterized the products by FTIR, NMR, elemental, and mass analyses. The stability constants (KS) of the two derivatives with a series of acyclic and cyclic alcohols were calculated. The modified CDs could discriminate the nature of the opponent’s molecules [86]. Another study reported two other β -CD derivatives [87]. The novel permethylated β -CD was modified using naphthalene and quinolone fluorophores to form two derivatives. Inclusion mode, complex-induced fluorescent behavior, binding ability, and selectivity for bile salts of biological relevance were observed [88]. A convenient method was applied to synthesize two β -CD derivatives, which were modified by (1-naphthyloxamino)-ethyleneamino or (1-naphthyloxamino)-diethylenediamino moiety; a high product yield was obtained. Liu et al. evaluated the inclusion ability of the derivatives and reported that a series of fluorescent dyes could form inclusion complexes. The two derivatives had satisfactory molecular recognition ability [89].

Martina et al. synthesized novel water-soluble β -aminoalcohol β -CD derivatives via the reactions of nucleophilic epoxide opening. The derivative was linked by aminoalcohol subunits and showed improved solubility. The reaction condition was optimized through microwave irradiation, and a high product yield was obtained [90].

L-Carnosine (LCar) is one of the most widely distributed copper(II)-endogenous dipeptides. Although the physiological role of LCar remains unclear, many functions of this compound have been proposed. LCar has an important pathological role by reducing or preventing the disturbance of metal ions. The potential therapeutic applications of LCar are limited because of the hydrolysis of specific dipeptidase (carnoseptidase). D-Carnosine (DCar) is a naturally occurring dipeptide enantiomer that exhibits the same properties as those of LCar; that is, they cannot be hydrolyzed enzymatically. DCar is a promising chemical modification of LCar for reducing enzymatic hydrolysis and conjugation with carbohydrate moieties to improve the tissue specificity of transport and enhance the bioavailability of the peptide. DCar was functionalized with β -CD and characterized by NMR. Similar LCar derivatives exhibit differences particularly with respect to dimeric species. Based on spectroscopic data, this stereoselectivity was driven by noncovalent interactions, such as hydrogen bonding, CH-π interaction, and spatial and hydrophobic effects of the CD cavity [91].

When β -CD is modified with a natural product, some properties of the raw product might change. Yang et al. successfully prepared many scutellarin-β -CD derivatives that were covalently bound. The water solubility test demonstrated that the aqueous solubility, stability, and cytotoxicity of the conjugates were significantly higher than those of scutellarin. The high antitumor activity and stability of the water-soluble conjugates indicate their potential for use in chemotherapy for human colon cancer [92]. Li et al. found that phosphate served as a catalyst in the synthesis of β -CD derivatives, which are connected to maleic and itaconic acids. They also studied the reaction conditions and found that the esterification rates of β -CD-maleic and β -CD-itaconic acids were 70.38% and 21.02%, respectively [93].

To deal with continuous changes and widen the application of CD in many aspects, Moutard et al. synthesized new phospholipidyl-β -CD derivatives, which exhibited satisfactory self-organization properties. However, the NMR experiments could not rapidly assess the clearly assigned structure and purity of the final product. Therefore, ESI-MS was combined with MS/MS to identify new amphiphilic compounds [94]. The derivatives could serve as artificial enzymes. To solve this problem, Bjerre et al. obtained β -CD derivatives containing trifluoromethyl groups at C-6 and discovered that trifluoromethylated alcohol was an artificial enzyme [95].

Takenaka et al. prepared a β -CD derivative connected to a naphthalene fluorophore. They investigated the stoichiometric binding of the derivative and alcohol guest molecules through fluorescent titration. The binding constant of the 1:1 host–guest inclusion complexes was dependent on alcohol molecules. Hence, the β -CD cavity was involved in guest sensing, and the partially self-included naphthalene probe of the derivative would extrude from the cavity upon binding to the guest molecules. According to the geometry of the inclusion complexes and energy changes, van der Waals interactions between the host and guest played an important role in fit-induced inclusion [96]. Wang et al. modified vinylene-functionalized cationic β -CD with vinylized silica and used it as a CSP to separate compounds [97].

Hydroxyl groups could become additional functionalized groups. Zhang et al. synthesized many β -CD derivatives, which were modified using the anthracene moiety and exhibited good solubility. They also studied the fluorescence properties of these derivatives [98].

Casas-Solvas et al. used two approaches for obtaining β -CD derivatives bearing azobenzene in the C- 6 position. Furthermore, a convenient click chemistry method was applied to produce 1,2,3-triazole-linked azobenzene-CD derivatives [99]. Alvarez-Dorta et al. explored a method for modifying the primary face of CDs [100].

### 3.2. Multiple substitutions of CDs

**Figure 6:**
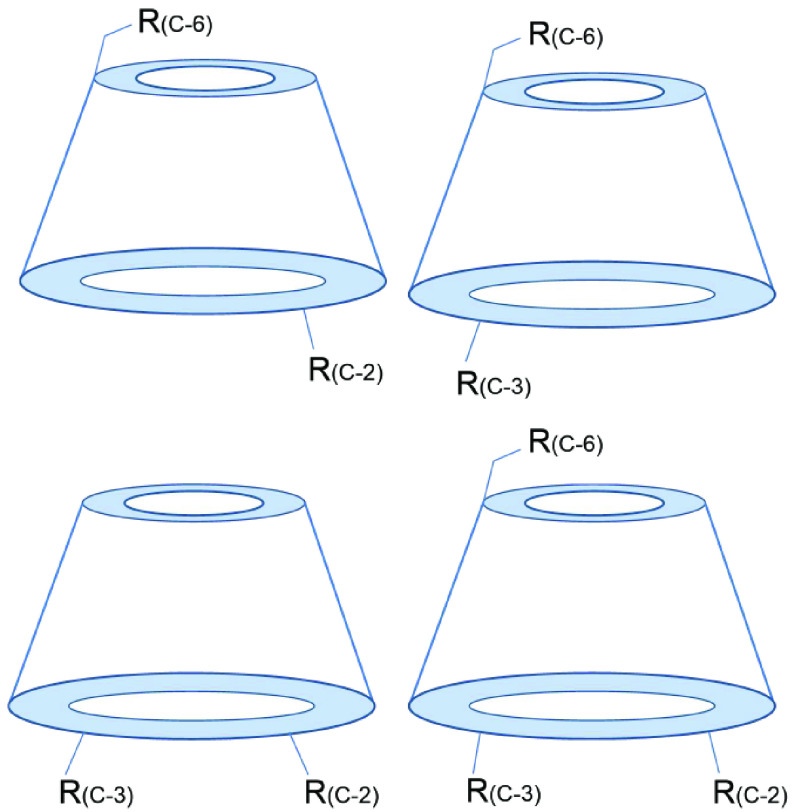
The types of multiple replaced CD derivatives.

When the two-hydroxyl groups of CD are substituted to form corresponding derivatives, they are usually based on different reactivities. Furthermore, a protection/deprotection method is usually employed to obtain the desired derivative. The three-hydroxyl groups of CD exist when they are simultaneously substituted. The structures of these derivatives are shown in Figure 6.

#### 3.2.1. Displacement of two and three positions

The C-2 and C-3 hydroxyls of CDs are located on the secondary face. The reaction is generally carried out by substituting the two-position first and further reacting with the three-position.

Menuel et al. applied mechanical lapping to modify different CDs at room temperature. Mono-2-tosylated CDs were obtained in good yield and in solid state. The yield was found to be dependent on the nature of the base and reaction time. By using NMR spectroscopy, they found that the highest yields of mono-2-tosyl CDs were obtained using KOH as a base and within a short reaction time. Lastly, mono-(2,3-manno-epoxide)-CDs were synthesized by mechanical mixing. The modified CDs were characterized using many technologies. The reaction mechanism was provided by the supramolecular arrangement of molecules in the solid state [25]. Fujita et al. obtained 2,3-substituted CDs as the main product [101]. Xiao et al. synthesized bis-de-O-methylated α- CD derivatives and reported the corresponding mechanism. In this reaction, the use of DIBAL-H as a chemical ‘scalpel’ showed that some parts of CD might be involved in the reaction [102].

#### 3.2.2. Displacement of two and six positions

Given that the C-2 and C-6 hydroxyl groups of CDs are located on different faces, this technology is difficult to use. However, some researchers obtained certain derivatives. Zhou and Zeng used Boger’s method [103] to prepare (2,6-di-O-methyl)-β -CD (DM-β -CD). They blended DM-β -CD with hydroxy-terminated silicone oil, prepared coated solid-phase microextraction fiber through sol-gel technology, and applied it to headspace solidphase microextraction for analysis of ephedrine and methamphetamine in human urine by gas chromatography. The derivative exhibited the advantages of the unique cavity-shaped cyclic molecular structure of CD and the superiorities of the sol-gel-coating technique. The derivative displayed satisfactory extraction ability and operational stability. Under the optimum conditions, the proposed headspace SPME-GC method had good linearity and satisfactory recovery rate [104].

#### 3.2.3. Displacement of three and six positions

Limited studies have been conducted on the modification of the three and six positions. Baudin et al. prepared per(3,6-anhydro)CD derivatives. In their subsequent study, they found a novel derivative that could produce new lanthanide chelates by carefully selecting the size and functional groups [105].

#### 3.2.4. Displacement of two, three, and six positions

Derivatives also possess improved properties when the hydroxyl at different positions is superseded by other groups. Three CD derivatives were successfully obtained by Bicchi et al. A previous study discovered that some CD derivatives might have drawbacks. The results of these experiments motivated these scholars to search for additional CD derivatives. Furthermore, 2,6-dimethyl-3-pentyl-β -CD and 2,3,6-tripentyl-β -CD were used in capillary GC, and 10 compounds were separated. Among different derivatives that CDs can produce, one is based on their average degree of substitution [106]. Junge et al. prepared highly enantioselective CD derivatives by exchanging a methyl group for an acetyl substituent in a single glucose [107].

Ma et al. synthesized a range of SBEβ -CD with a mean degree of substitution (DS) and examined the products using several technologies. The substitution reaction occurred simultaneously at C-6 and C- 2 in the moderate alkaline solution, and the proportion of C-2 substitution increased with increasing DS. The DS of SBE-β -CDs demonstrated good reproducibility, and analysis by capillary electrophoresis showed the normal distribution of the composition [108]. A new β -CD derivative was designed and synthesized by Zhang et al. by substituting different positions of CDs with different groups. The novel derivative was called carboxymethyhydroxypropyl-β -CD. DTF, IR, and NMR methods were used to verify the derivative. The ability of the derivative to bind to insulin and its cytotoxicity in Caco-2 cells were investigated. The derivative could form an inclusion complex with insulin and obtained better cytotoxicity than other β -CD derivatives. Hence, the developed derivative might be a promising protein carrier for oral delivery of protein drugs [109].

The selective modification of CDs on definite positions is dominant in CD modification. The method could be used to obtain specific structures of CD derivatives for subsequent study of their properties. Future studies should explore other applications of CDs that have accurate structures.

## 4. Random modifications of CDs

**Figure 7:**
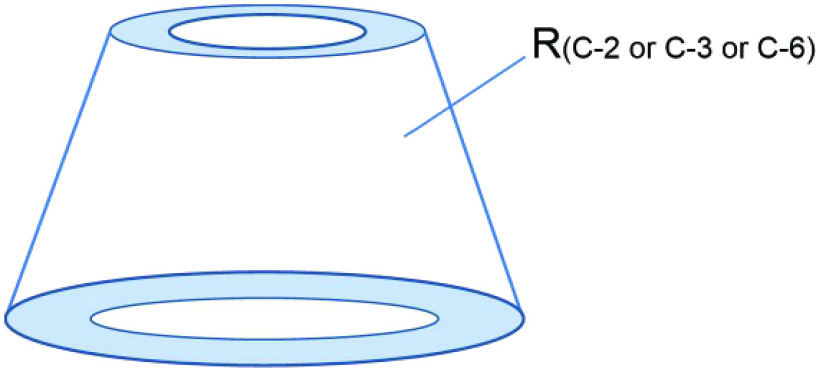
The structures of the randomly substituted CD derivatives.

Randomly substituted CD derivatives are modified at various positions and confirmed by their DS. The exact structure and ratio of these CD derivatives are unknown (Figure 7). Moreover, CD derivatives have potential for widespread commercial applications. In future works, inexpensive and randomly substituted CD derivatives should be developed.

Randomly substituted CD derivatives can also be used as CSPs. Berthod et al. synthesized CD derivatives that can be used in capillary gas chromatography. These derivatives had good solubility in water and were able to separate 13 trifluoroacetylated sugars. Most of the sugars had their enantiomeric elution order. As CSPs, these derivatives had a wide range of usage temperatures [110]. Zhong et al. developed a new β -CD derivative, which was modified with dinitrophenyl, and used it as a CSP for chiral separation of analytes. This study is the first report of dextrin, which contains π-electron-deficient substituents. Many factors can affect the ability of CSPs [111].

Cucinotta et al. observed that a capped CD derivative, namely hemispherodextrin, could be applied as an efficient chiral separation reagent for many phenoxyacid enantiomeric pairs. The derivative exhibited good separation behavior [112].

To separate neutral racemates, Tanaka et al. prepared five different anionic CD derivatives as chiral selectors and used them to successfully separate 40 basic racemates [113]. Zhou et al. used a β -CD tosylate derivative as a CSP to separate tetracyclines. These derivatives had satisfactory stability and reproducibility [114]. Xu et al. used CD derivatives to separate enantiomers and explored separation conditions, such as pH, temperature, and running voltage [115]. Issaraseriruk et al. applied heptakis(2,3-di-O-methyl-6-O-tert-butyldimethylsilyl)-β -CD to analyze trifluoroacetyl-derivatized 1-phenylalkylamines with different types and positions of the substituent [116].

Although certain CD derivatives have separation ability, they have other abilities, such as good water solubility and photostability. Resveratrol functionalized CD to form a new derivative, which exhibited better photostability than resveratrol [117]. Pitha et al. converted α-, β -CD and its hydroxypropyl derivative into the corresponding sulfates in chlorosulfonic acid to enhance drug solubility [118]. Shao et al. reported that CD derivatives could be used as transdermal absorption enhancers for insulin [119]. Cheng et al. used many technologies to characterize the structure of succinic-β -CD as a potential emulsion stabilizer. The derivative was more stable than β -CD after 24 h of storage at 25 °C [120].

## 5. Reconstruction of the skeleton of CDs

**Figure 8:**
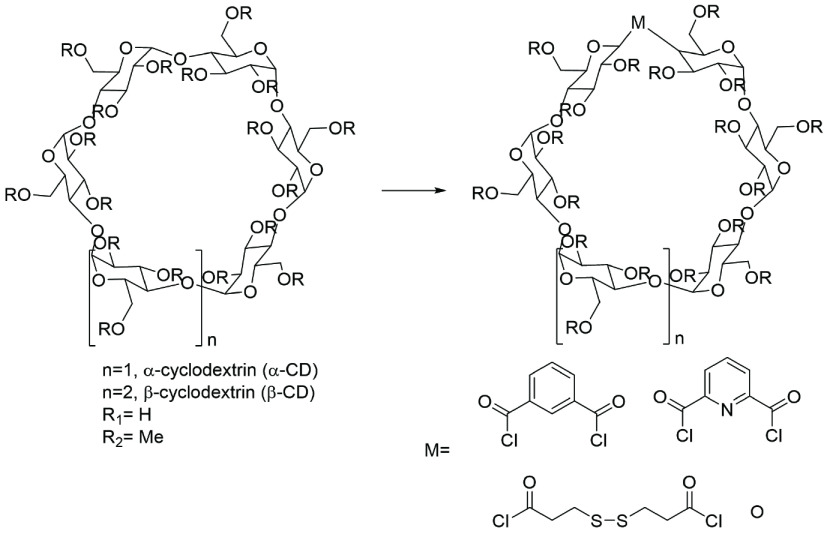
The structures of derivatives modified by changes of the skeleton.

Changing the skeleton structure of cyclodextrin is a new theory, which could form a new cavity of derivatives and have innovative application. The structures of derivatives are given in Figure 8.

Kida developed new CD derivatives, which were connected by β -(1,4)-glucosidic bonds. The derivatives displayed novel inclusion ability and selectivity [121–123].

Kikuzawa et al. modified the α-(1,4)-glucosidic bond to a β -(1,4)-glucosidic bond and studied their inclusion ability. The derivative, as a host molecule, demonstrated inclusion selectivity for sodium p-nitrobenzoate, in contrast to a permethylated CD. However, the host molecule of the derivative exhibited para-isomer selectivity towards the sodium p-nitrophenolate guest. Hence, the inclusion selectivity was affected by the properties of guest molecules. The structures of the complexes of the β -(1,4)-type CD derivatives with different kinds of nitrobenzoates were determined by 1 H NMR studies [124]. In addition, novel stimulus-responsive CDs modified by a disulfide unit were successfully obtained. The inclusion ability of the derivatives was regulated by opening and closing of the ring through dithiol-disulfide interconversion [125].

The method of reconstituting the CD skeleton is a new path for modifying CDs in recent years. Moreover, these new derivatives have different properties compared to the original CDs. This new method may have broader use in the future.

## 6. Conclusions

Since the discovery of CDs, research on this area has progressed significantly. CDs are applied in many fields, such as pharmaceuticals, biomedicine, textiles, and separation. Many CD derivatives have been commercially available for many years, but their prices are still not accepted by most people. At present, most derivatives have been limited to laboratory synthesis. This review summarizes different types of CD substitution and the use of certain derivatives in separation of isomers. The structure of CDs can be further improved by modifying the different structures and reaction conditions of CD derivatives to benefit ecologically sustainable chemical processes and technologies. Scholars have substantial interest in the practical application of CD derivatives and supramolecular chemistry based on structural modification. In the future, potential host–guest systems, new polymer materials, and utilization in clinical treatments should be explored based on the novel structure of CD derivatives.

## References

[1] (1998). general overview of cyclodextrin chemistry. Chemical Reviews.

[2] (2014). Analytical techniques for characterization of cyclodextrin complexes in aqueous solution: a review. Journal of Pharmaceutical and Biomedical Analysis.

[3] (2018). Reactivity and Analysis.

[4] (2014). Review: A history of cyclodextrins. Chemical Reviews.

[5] (2013). A novel biodegradable $\beta $-cyclodextrin-based hydrogel for the removal of heavy metal ions. Carbohydrate Polymers.

[6] (2013). International Journal of Pharmaceutics.

[7] (2014). Complex macromolecular architecture design via cyclodextrin host/guest complexes. Progress in Polymer Science.

[8] (2009). aacute;ndara J. A review on the use of cyclodextrins in foods. Food Hydrocolloids.

[9] (2006). Flavour encapsulation and controlled release-a review. International Journal of Food Science {\&} Technology.

[10] (2019). Characterization of in vitro and in vivo bioactivity of a ferulic acid-2-Hydroxypropyl-$\beta $-cyclodextrin inclusion complex. Colloids and Surfaces B: Biointerfaces.

[11] (2008). Protein nanoparticles as drug carriers in clinical medicine. Advanced Drug Delivery Reviews.

[12] (2012). Determination of hydroxy acids in cosmetics by chemometric experimental design and cyclodextrin-modified capillary electrophoresis. Electrophoresis.

[13] (2019). Solubilization of ultraviolet absorbers by cyclodextrin and their potential application in cosmetics. Journal of Inclusion Phenomena and Macrocyclic Chemistry.

[14] (1982). Cyclodextrins in Foods, Cosmetics and.

[15] (2019). Hemp-based adsorbents for sequestration of metals: a review. Environmental Chemistry Letters.

[16] (2011). Cyclodextrin-based inclusion complexation bridging supramolecular chemistry and macromolecular self-assembly. Chemical Society Reviews.

[17] (2017). Collaborative routes to clarifying the murky waters of aqueous supramolecular chemistry. Nature Chemistry.

[18] (2009). Cyclodextrin-based supramolecular polymers. Chemical Society Reviews.

[19] (2009). Enzymes from solvent-tolerant microbes: useful biocatalysts for non-aqueous enzymology. Critical Reviews in Biotechnology.

[20] (1991). Ballesteros A. Applied Biochemistry and Biotechnology.

[21] (2012). Recent development of cyclodextrin chiral stationary phases and their applications in chromatography. Journal of Chromatography A.

[22] (1990). Derivatized cyclodextrins for normal-phase liquid chromatographic separation of enantiomers. Analytical Chemistry.

[23] (2019). -hydroxyphenylacetic acid with cyclodextrins as an effective phase-transfer catalyst and its reaction mechanism. Turkish Journal of Chemistry.

[24] (2015). Supramolecular catalysis in metal-ligand cluster hosts. Chemical Reviews.

[25] (2016). Cyclodextrins as effective additives in AuNP-catalyzed reduction of nitrobenzene derivatives in a ball-mill. Green Chemistry.

[26] (2017). Cyclodextrin nanosponges: a potential catalyst and catalyst support for synthesis of xanthenes. Research on Chemical Intermediates.

[27] (2018). A manganese porphyrin-$\alpha $-cyclodextrin conjugate as an artificial enzyme for the catalytic epoxidation of polybutadiene. Chemical Communications.

[28] (2003). Synthesis, NMR spectroscopic characterization and polysiloxane-based immobilization of the three regioisomeric monooctenylpermethyl-$\beta $-cyclodextrins and their application in enantioselective GC. European Journal of Organic Chemistry.

[29] (1991). Chemospecific manipulations of a rigid polysaccharide: syntheses of novel chitosan derivatives with excellent solubility in common organic solvents by regioselective chemical modifications. Macromolecules.

[30] (2019). Synthesis of substituted cyclodextrins. Environmental Chemistry Letters.

[31] (2000). Selectively monomodified cyclodextrins. Synthetic strategies. Journal of Organic Chemistry.

[32] (2010). Synthesis of a $\beta $-cyclodextrin derivate and its molecular recognition behavior on modified glassy carbon electrode by diazotization. Tetrahedron.

[33] (1998). Methods for selective modifications of cyclodextrins. Chemical Reviews.

[34] (2007). Cyclodextrins and their pharmaceutical applications. International Journal of Pharmaceutics.

[35] (1997). Utilization of cyclodextrins in industrial products and processes. Journal of Materials Chemistry.

[36] (2011). Cyclodextrin-based inclusion complexation bridging supramolecular chemistry and macromolecular self-assembly. Chemical Society Reviews.

[37] (2014). Cyclodextrin-based supramolecular assemblies and hydrogels: recent advances and future perspectives. Macromolecular Rapid Communications.

[38] (2014). Engineering responsive polymer building blocks with host-guest molecular recognition for functional applications. Accounts of Chemical Research.

[39] (2017). Dynamic macromolecular material design-the versatility of cyclodextrin-based host-guest chemistry. Angewandte Chemie International Edition.

[40] (2019). Cyclodextrin-based sustained gene release systems: a supramolecular solution towards clinical applications. Materials Chemistry Frontiers.

[41] (2010). Cyclodextrin-complexed curcumin exhibits anti-inflammatory and antiproliferative activities superior to those of curcumin through higher cellular uptake. Biochemical Pharmacology.

[42] (2014). -cyclodextrin inclusion complex: stability, solubility, characterization by FT-IR, FT-Raman, X-ray diffraction and photoacoustic spectroscopy, and food application. Food Chemistry.

[43] (2012). Inclusion complex of novel curcumin analogue CDF and $\beta $-cyclodextrin (1: 2) and its enhanced in vivo anticancer activity against pancreatic cancer. Pharmaceutical Research.

[44] (2013). Solubilities of quercetin in three $\beta $-cyclodextrin derivative solutions at different temperatures. Journal of Molecular Liquids.

[45] (1994). Cyclodextrins as building blocks for supramolecular structures and functional units. Angewandte Chemie International Edition.

[46] (2006). Application of nanoparticles in electrochemical sensors and biosensors. Electroanalysis.

[47] (2013). Enantioselective sorption of some chiral carboxylic acids by various cyclodextrin-grafted iron oxide magnetic nanoparticles. Tetrahedron Asymmetry.

[48] (2010). Photocontrolled size changes of doubly-threaded dimer based on an $\alpha $-cyclodextrin derivative with two recognition sites. Chemistry Letters.

[49] (2008). Synthesis of a $\beta $-cyclodextrin derivative bearing an azobenzene group on the secondary face. Tetrahedron Letters.

[50] (1986). Hydrophilic cyclodextrin derivatives enable effective oral administration of steroidal hormones. Journal of Pharmaceutical Sciences.

[51] (2005). New cyclomaltoheptaose ($\beta $-cyclodextrin) derivative 2-O-(2-hydroxybutyl) cyclomaltoheptaose: preparation and its application for the separation of enantiomers of drugs by capillary electrophoresis. Carbohydrate Research.

[52] (2008). Synthesis of a novel $\beta $-cyclodextrin derivative with high solubility and the electrochemical properties of ferrocene-carbonyl-$\beta $-cyclodextrin inclusion complex as an electron transfer mediator. Electrochemistry Communications.

[53] (2003). Enantioselective recognition of aliphatic amino acids by $\beta $-cyclodextrin derivatives bearing aromatic organoselenium moieties on the primary or secondary side. European Journal of Organic Chemistry.

[54] (2009). Regioselective access to 3I-O-substituted-$\beta $-cyclodextrin derivatives. Chemical Communications.

[55] (2017). Djeda\"ıni-. Organic {\&} Biomolecular Chemistry.

[56] (1989). Marked differences in molecular association behavior between two regioisomers of $\gamma $-cyclodextrin derivatives bearing a pyrenecarbonyl moiety at C-2 and C-3. Chemistry Letters.

[57] (1986). Regiospecific sulfonation onto C-3 hydroxyls of beta-cyclodextrin. Preparation and enzyme-based structural assignment of 3A,3C and 3A3D disulfonates. Journal of the American Chemical Society.

[58] (1996). Selective modification at the 3-position of $\beta $-cyclodextrin. Tetrahedron Letters.

[59] (2003). -anhydrocyclodextrins. Functionalization of cyclodextrins via reactions of 2.

[60] (2008). Branched supramolecular polymers formed by bifunctional cyclodextrin derivatives. Tetrahedron.

[61] (2005). Jind\v{r}ich J, Ti&scaron;lerov&aacute; I. Simple preparation of 3'-O-substituted $\beta $-cyclodextrin derivatives using cinnamyl bromide. Journal of Organic Chemistry.

[62] (2018). Amino cyclodextrin per-O-methylation: synthesis of 3-monoamino-permethylated derivatives. Tetrahedron Letters.

[63] (2007). Efficient regioselective functionalizations of cyclodextrins carried out under microwaves or power ultrasound. Tetrahedron Letters.

[64] (1994). First selective synthesis of thio-$\beta $-cyclodextrin derivatives by a direct Mitsunobu reaction on free $\beta $-cyclodextrin. Tetrahedron Letters.

[65] (2013). The incorporation of calix[6]arene and cyclodextrin derivatives into sol-gels for the preparation of stationary phases for gas chromatography. Journal of Chromatography A.

[66] (1994). Enantiomer separation using mobile and immobile cyclodextrin derivatives with electromigration. Electrophoresis.

[67] (2012). Chemically bonded cationic $\beta $-cyclodextrin derivatives and their applications in supercritical fluid chromatography. Journal of Chromatography A.

[68] (2012). Chemically bonded cationic $\beta $-cyclodextrin derivatives as chiral stationary phases for enantioseparation applications. Tetrahedron Letters.

[69] (2008). Click chemistry for facile immobilization of cyclodextrin derivatives onto silica as chiral stationary phases. Tetrahedron Letters.

[70] (2005). Kinetic control of threading of cyclodextrins onto axle molecules. Journal of the American Chemical Society.

[71] (2007). Face-selective [2]- and [3]rotaxanes: kinetic control of the threading direction of cyclodextrins. Chemistry-A European Journal.

[72] (2011). Photoresponsive formation of pseudo[2]rotaxane with cyclodextrin derivatives. Organic Letters.

[73] (2007). New cup-shaped $\alpha $-cyclodextrin derivatives and a study of their catalytic properties in oxidation reactions. Tetrahedron.

[74] (2014). -cyclodextrin derivatives: synthesis, cellular toxicity, and phase-solubility study. Carbohydrate Research.

[75] (2017). The effect of urea moiety in amino acid binding by $\beta $-cyclodextrin derivatives: a 1000-fold increase in efficacy comparing to native $\beta $-cyclodextrin. Carbohydrate Polymers.

[76] (2018). \beta $-Cyclodextrin-functionalized chitosan/alginate compact polyelectrolyte complexes (CoPECs) as functional biomaterials with anti-inflammatory properties. ACS Applied Materials {\&} Interfaces.

[77] (1995). A general method for the synthesis of cyclodextrinyl aldehydes and carboxylic acids. Journal of Organic Chemistry.

[78] (2012). A novel synthetic strategy for monosubstituted cyclodextrin derivatives. Chemical Communications.

[79] (2011). Enantiomeric separation in high-performance liquid chromatography using novel $\beta $-cyclodextrin derivatives modified by R-configuration groups as chiral stationary phases. Talanta.

[80] (2010). and evaluation of chiral monolithic column modified by $\beta $-cyclodextrin derivatives. Talanta.

[81] (2013). Hollow nanosphere fabricated from $\beta $-cyclodextrin-grafted $\alpha $,$\beta $-poly(aspartic acid) as the carrier of camptothecin. Colloids and Surfaces B.

[82] (2019). Efficacy of polyurethane graft on cyclodextrin to control drug release for tumor treatment. Journal of Colloid and Interface Science.

[83] (2007). Inhibition of \textit{S. aureus} $\alpha $-hemolysin and \textit{B. anthracis} lethal toxin by $\beta $-cyclodextrin derivatives. Bioorganic {\&} Medicinal Chemistry.

[84] (2005). A water-soluble $\beta $-cyclodextrin derivative possessing a fullerene tether as an efficient photodriven DNA-cleavage reagent. Tetrahedron Letters.

[85] (2001). -cyclodextrin derivative bearing a cyclohexylamino moiety and its inclusion complexation with organic dye molecules. Microchemical Journal.

[86] (1999). Molecular recognition studies on supramolecular systems. 22. Size, shape, and chiral recognition of aliphatic alcohols by organoselenium-modified cyclodextrins. Journal of Organic Chemistry.

[87] (1999). Enantioselective recognition of aliphatic amino acids by organoselenium modified $\beta $-cyclodextrins. Supramolecular Chemistry.

[88] (2007). -cyclodextrin derivatives appended with chromophores as efficient fluorescent sensors for the molecular recognition of bile salts. Journal of Organic Chemistry.

[89] (2002). Synthesis of novel $\beta $-cyclodextrin derivatives bearing a 1-naphthyloxamino-oligo(ethyleneamino) moiety and their inclusion complexation with some fluorescent dyes. Supramolecular Chemistry.

[90] (2011). Synthesis of water-soluble multidentate aminoalcohol $\beta $-cyclodextrin derivatives via epoxide opening. Carbohydrate Research.

[91] (2011). Noncovalent interaction-driven stereoselectivity of copper(II) complexes with cyclodextrin derivatives of L- and D-carnosine. Inorganic Chemistry.

[92] (2013). Scutellarin-cyclodextrin conjugates: Synthesis, characterization and anticancer activity. Carbohydrate Polymers.

[93] (2015). Synthesis of two $\beta $-cyclodextrin derivatives containing a vinyl group. Carbohydrate Research.

[94] (2003). Structural identification of new glycolipids based on cyclodextrin using high-resolution positive and negative electrospray ionization mass spectrometry. Rapid Communications in Mass Spectrometry.

[95] (2007). Synthesis of some trifluoromethylated cyclodextrin derivatives and analysis of their properties as artificial glycosidases and oxidases. European Journal of Organic Chemistry.

[96] (2007). -cyclodextrin derivative as a receptor for various types of alcohols having cyclic and macrocyclic rings. Journal of Molecular Structure.

[97] (2012). Chemically bonded cationic $\beta $-cyclodextrin derivatives and their applications in supercritical fluid chromatography. Journal of Chromatography A.

[98] (2006). Syntheses and photophysical studies of cyclodextrin derivatives with two proximate anthracenyl groups. Journal of Organic Chemistry.

[99] (2008). -cyclodextrin derivatives functionalized with azobenzene. Tetrahedron.

[100] (2015). 'ın I et al. Angewandte Chemie International Edition.

[101] (1987). Synthesis of specifically modified maltooligosaccharides by enzymic degradation of cyclodextrin derivatives. Substrate-based investigation of the active site of Taka-amylase. Journal of Organic Chemistry.

[102] (2013). Synthesis of four mono-functionalized $\alpha $-cyclodextrin derivatives for further confirming DIBAL-H-promoted bis-de-O-methylation mechanism. Tetrahedron.

[103] (1978). Cyclodextrin chemistry. Selective modification of all primary hydroxyl groups of $\alpha $- and $\beta $-cyclodextrins. Helvetica Chimica Acta.

[104] (2006). Novel fiber coated with $\beta $-cyclodextrin derivatives used for headspace solid-phase microextraction of ephedrine and methamphetamine in human urine. Analytica Chimica Acta.

[105] (2005). Ionic complexation properties of per(3,6-anhydro) cyclodextrin derivatives towards lanthanides. Carbohydrate Research.

[106] (1992). Cyclodextrin derivatives in the GC separation of racemic mixtures of volatile compounds. Part V: Heptakis.

[107] (2003). Selectivity tuning of cyclodextrin derivatives by specific substitution. Journal of Separation Science.

[108] (2016). The synthesis and process optimization of sulfobutyl ether $\beta $-cyclodextrin derivatives. Tetrahedron.

[109] (2013). Synthesis and evaluation of a novel $\beta $-cyclodextrin derivative for oral insulin delivery and absorption. International Journal of Biological Macromolecules.

[110] (1990). Chiral recognition of racemic sugars by polar and nonpolar cyclodextrin-derivative gas chromatography. Carbohydrate Research.

[111] (2006). Development of dinitrophenylated cyclodextrin derivatives for enhanced enantiomeric separations by high-performance liquid chromatography. Journal of Chromatography A.

[112] (2001). Hemispherodextrins, a new class of cyclodextrin derivatives, in capillary electrophoresis. Journal of Chromatography A.

[113] (1996). Separation of neutral and basic enantiomers by cyclodextrin electrokinetic chromatography using anionic cyclodextrin derivatives as chiral pseudo-stationary phases. Journal of High Resolution Chromatography.

[114] (2017). \beta $-Cyclodextrin-ionic liquid polymer based dynamically coating for simultaneous determination of tetracyclines by capillary electrophoresis. Electrophoresis.

[115] (2003). -allenoic acid by capillary zone electrophoresis using cyclodextrin derivatives. Chiral separation of 2.

[116] (2018). Substituent effects on chiral resolutions of derivatized 1-phenylalkylamines by heptakis(2,3-di-. Chirality.

[117] (2018). -cyclodextrin: synthesis, characterization, and photostability. Journal of Chemistry.

[118] (1991). Cyclodextrin sulfates: characterization as polydisperse and amorphous mixtures. Pharmaceutical Research.

[119] (1994). Cyclodextrins as mucosal absorption promoters of insulin. II. Effects of $\beta $-cyclodextrin derivatives on $\alpha $-chymotryptic degradation and enteral absorption of insulin in rats. Pharmaceutical Research.

[120] (2017). Preparation and properties of octenyl succinate $\beta $-cyclodextrin and its application as an emulsion stabilizer. Food Chemistry.

[121] (2005). Synthesis of novel cyclodextrin derivatives by aromatic spacer insertion and their inclusion ability. Tetrahedron.

[122] (2003). A facile synthesis of novel cyclodextrin derivatives incorporating one $\beta $-(1,4)-glucosidic bond and their unique inclusion ability. Chemical Communications.

[123] (2002). A facile synthesis of novel types of cyclodextrin derivatives by insertion of an aromatic dicarbonyl spacer into a permethylated $\alpha $-cyclodextrin skeleton. Chemical Communications.

[124] (2005). Short synthesis of skeleton-modified cyclodextrin derivatives with unique inclusion ability. Journal of Organic Chemistry.

[125] (2007). Synthesis of stimuli-responsive cyclodextrin derivatives and their inclusion ability control by ring opening and closing reactions. Organic Letters.

